# Temperature Sensitive Mutations in Influenza A Viral Ribonucleoprotein Complex Responsible for the Attenuation of the Live Attenuated Influenza Vaccine

**DOI:** 10.3390/v10100560

**Published:** 2018-10-15

**Authors:** Luis Martínez-Sobrido, Olve Peersen, Aitor Nogales

**Affiliations:** 1 Department of Microbiology and Immunology, University of Rochester School of Medicine and Dentistry, 601 Elmwood Avenue, Rochester, New York, NY 14642, USA; 2 Department of Biochemistry and Molecular Biology, Colorado State University, Fort Collins, Colorado, CO 80523, USA; Olve.Peersen@colostate.edu

**Keywords:** influenza virus, influenza vaccine, live-attenuated influenza virus, recombinant influenza virus, viral polymerase complex, nucleoprotein, temperature-sensitive, cold-adapted, attenuated

## Abstract

Live attenuated influenza vaccines (LAIV) have prevented morbidity and mortality associated with influenza viral infections for many years and represent the best therapeutic option to protect against influenza viral infections in humans. However, the development of LAIV has traditionally relied on empirical methods, such as the adaptation of viruses to replicate at low temperatures. These approaches require an extensive investment of time and resources before identifying potential vaccine candidates that can be safely implemented as LAIV to protect humans. In addition, the mechanism of attenuation of these vaccines is poorly understood in some cases. Importantly, LAIV are more efficacious than inactivated vaccines because their ability to mount efficient innate and adaptive humoral and cellular immune responses. Therefore, the design of potential LAIV based on known properties of viral proteins appears to be a highly appropriate option for the treatment of influenza viral infections. For that, the viral RNA synthesis machinery has been a research focus to identify key amino acid substitutions that can lead to viral attenuation and their use in safe, immunogenic, and protective LAIV. In this review, we discuss the potential to manipulate the influenza viral RNA-dependent RNA polymerase (RdRp) complex to generate attenuated forms of the virus that can be used as LAIV for the treatment of influenza viral infections, one of the current and most effective prophylactic options for the control of influenza in humans.

## 1. Influenza Viruses and Their Impact on Human Health

Influenza viruses are enveloped viruses belonging to the *Orthomyxoviridae* family and are the causative agents of influenza. There are four recognized influenza types denoted as A, B, C, and the recently discovered D [1,2,3,4,5]. Influenza A viruses (IAV) are able to infect a wide range of avian and mammalian species, including humans, and mostly exist in the wild aquatic fowl reservoir [6,7,8,9,10]. IAV have been associated with pandemics in humans, mainly because their ability to infect different hosts, reassort their genome, and freely replicate in the absence of pre-existing immunity in humans. In contrast, influenza B viruses (IBV) are mainly restricted and adapted to humans, although sporadic infections of seals have been documented [11]. Because of their restriction to infect humans, IBV have not been responsible, to date, for human pandemics. Influenza C viruses (ICV) normally cause a mild respiratory illness in humans and are not thought to cause epidemics or pandemics [12,13]. The newly identified influenza D viruses (IDV) primarily affect cattle and pigs and, to date, are not known to infect or cause illness in humans [2,3,14]. IAV and IBV infections represent a serious public health problem that cause contagious respiratory disease and substantial morbidity and mortality in humans, resulting in a considerable economic burden worldwide [15,16,17]. Public health concerns posed by IAV and IBV in humans are further aggravated by their ability to efficiently transmit and the limited antiviral therapeutic options for the treatment of viral infections.

**Figure 1:**
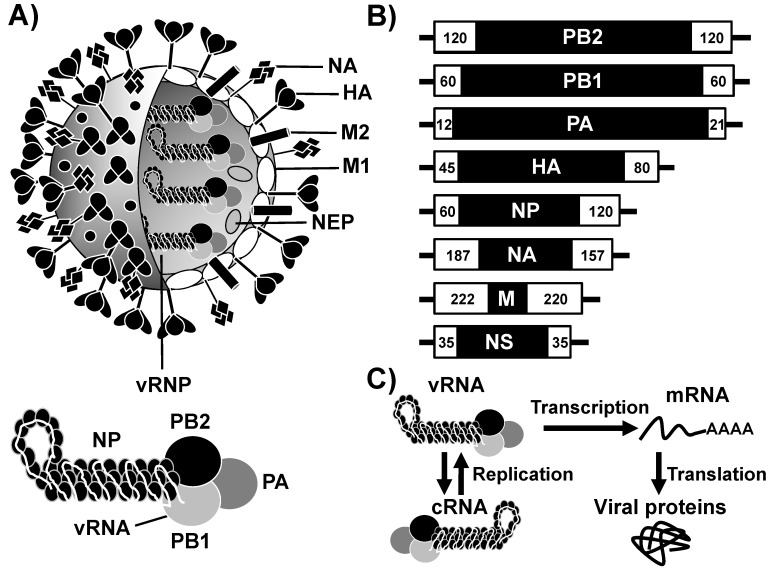
Influenza A virion structure, genome organization, and viral replication and transcription. (**A**) Virion structure: IAV are surrounded by a lipid bilayer containing the two viral glycoproteins hemagglutinin (HA) and neuraminidase (NA). Also, in the virion membrane is the ion channel matrix 2 (M2) protein. Under the viral lipid bilayer is a protein layer composed of the matrix 1 (M1) protein and the nuclear export protein (NEP). Inside the virion are the eight viral (v)RNA segments that are encapsidated by the viral nucleoprotein (NP) as viral ribonucleoprotein (vRNP) complexes. Associated with each vRNP is the viral RNA-dependent RNA polymerase (RdRp) complex made of the three polymerase subunits PB2, PB1, and PA that, together with the viral NP are the minimal components required for viral replication and transcription. Viral proteins in the virion particle (top) and in the vRNP (bottom) are indicated. The viral proteins showed in the illustration of the viral particle do not maintain the stoichiometric that is found in a mature virion. (**B**) Genome organization: IAV contain eight single-stranded, negative-sense, viral RNA segments (PB2, PB1, PA, HA, NP, NA, M, and NS). Each viral segment contains non-coding regions (NCR) at the 3′ and 5′ ends (black lines). Also, at the 3′ and 5′ end of the viral RNAs are the packaging signals, responsible for the efficient encapsidation into nascent virions (white boxes). Numbers represent the nucleotide length of the 3′ or 5′ packaging signals in each of the viral RNA segments. (**C**) Viral genome replication, transcription and translation: IAV NP and the viral polymerase complex PB2, PB1, and PA associated with the viral RNA form the vRNP complexes that are responsible for viral replication and transcription. vRNP complexes initiate transcription from the viral promoters located within the NCR at the 3′ termini of each of the vRNAs. Transcription results in the synthesis of IAV mRNA that are translated to proteins. IAV polymerase complex is also involved in the replication of the vRNA into a complementary (c)RNA that serve as template for the amplification of vRNAs. Newly synthesized vRNPs, together with the structural viral proteins result in the formation of new IAV.

IAV are divided into multiple subtypes based on the two glycoproteins located on the surface of the virus, the hemagglutinin (HA) and the neuraminidase (NA) (Figure 1) [18]. The new generation and implementation of sequencing technologies, as well as the development of better bioinformatics tools, have helped to discover novel subtypes of IAV. Currently, there are 18 HA (H1 to H18) and 11 NA (N1 to N11) IAV subtypes [19,20]. However, only IAV H1N1 and H3N2 subtypes are presently circulating in humans. IAV are responsible of annual global epidemics, which are most effectively prevented through annual vaccination to reduce transmission and future infections [6,7,21,22]. The impact of IAV is further intensified by the occurrence of pandemics when novel viruses are introduced into the human population [23]. For instance, the first influenza pandemic of the 21st century was declared in 2009 after the emergence of a quadruple-reassortant swine-origin H1N1 IAV [22,23]. It is estimated that in about one year, the pandemic 2009 H1N1 IAV infected more than 600,000 individuals around the world, causing near 16,000 human deaths in over 200 countries [24].

On the other hand, IBV are divided into two major lineages that are co-circulating in the human population: the Victoria- and Yamagata-like lineages [21,25]. These two lineages diverged in the 1980s from the ancestral influenza B/Lee/40. Although perceived as less dangerous than IAV, IBV infections cause annual outbreaks of respiratory illness and are associated with excess morbidity and mortality in the pediatric population [26,27]. Typically, IBV epidemics are less severe than H3N2 IAV but more severe than H1N1 IAV in adults and the elderly population [28,29,30].

There are two major evolutionary mechanisms responsible for seasonal and pandemic influenza. First, antigenic drift, a characteristic shared with other RNA viruses, involves the introduction of mutations in the viral genome, leading to the selection of viral mutants with resistance against current antivirals and/or neutralizing antibodies (NAbs) [31,32,33]. Second, the segmented nature of IAV genome allow the exchange of viral segments between different viral strains co-infecting the same cell (antigenic shift). This reassortment event can lead to a new pandemic virus when a new virus is introduced in the human population. Given the ability of IAV to modify their genome and their rapid transmission, seasonal influenza vaccines need to be reformulated annually to ensure that the two viral glycoproteins (HA and NA) in the vaccine match circulating seasonal viruses. Therefore, even though vaccination is the best intervention option to protect against seasonal IAV infections, the efficiency of current vaccination approaches has been shown to be suboptimal for Influenza And new prophylactic approaches to protect against this important human virus are highly desirable [34,35].

Despite countermeasures against influenza that prevent (vaccines) or treat (antivirals) viral infections, this respiratory human pathogen infects 5–15% of the world population during seasonal epidemics. Notably, data from the World Health Organization (WHO) estimate that seasonal influenza virus infections are responsible of about 3–5 million cases of severe disease and about 250,000–650,000 deaths worldwide yearly [36] (http://www.who.int/news-room/fact-sheets/detail/influenza-(seasonal)). Current available options to counter influenza infections include both vaccines and antivirals. Vaccines are the most cost-effective approach, due to the induction of sterilizing immunity, and they are the primary prophylactic means to prevent influenza viral infections [35,37,38]. The first vaccine option is the chemically inactivated influenza vaccine (IIV), which requires a large quantity of virus to induce NAbs sufficient for protection against subsequent infections. However, IIV are suboptimal because the limited, if any, cellular-mediated immune responses they are able to induce. Moreover, immunocompromised and elderly individuals typically show reduced responses compared to young or healthy population [39,40]. Importantly, IIV has a limited protection when the seasonal vaccine formulation does not match the predicted circulating strains. Most recently, an influenza vaccine made of purified recombinant HA antigens (https://www.cdc.gov/flu/protect/vaccine/qa_flublok-vaccine.htm) has been licensed with similar immunogenic characteristics to IIV. The remaining prophylactic option is the live attenuated influenza vaccine (LAIV), which is associated with broader responses and protection by the induction of both innate and humoral B- and T-cell adaptive immune responses. In addition, its administration mimics the usual route of influenza virus infection that has been shown to provide more efficient cross-reactive cellular-mediated protection against heterologous influenza viruses, important in cases where the vaccine and circulating strains do not match [41,42]. However, current LAIV remain restricted for use in healthy children and non-pregnant adults.

Independent of type, seasonal influenza vaccines typically contain antigens from the two IAV subtypes (H1N1 and H3N2) and one IBV subtype (Victoria- and/or Yamagata-like lineages) currently circulating in humans [43,44]. Worryingly, over the past decade, seasonal influenza vaccines have shown a low protection efficacy rate [34,38,45]. In order to improve the effectiveness of current seasonal influenza vaccines, a quadrivalent influenza vaccine formulation, including both IBV lineage components (Victoria- and Yamagata-like lineages) was approved by the United States (US) Food and Drug Administration (FDA) for its use during the 2012 season [44]. However, from 2013 through 2016, the efficacy of the new quadrivalent LAIV in the US has been shown to be even lower than its predecessor, the trivalent formulation [46,47], leading the Advisory Committee on Immunization Practices (ACIP) to make the recommendation that the quadrivalent LAIV should not be used during 2016–17 or 2017–18 in the US [48,49]. However, for the current 2018–19 influenza season, LAIV will be included again among the eligible prophylactic options to prevent influenza infections (https://www.aafp.org/news/health-of-the-public/20180226acipmtg-laiv.html). The variability in influenza vaccine efficiency is leading to an increasing concern on how to combat influenza virus infection and protect population from seasonal infections. Consequently, new approaches are necessary to prevent the morbidity and mortality associated with influenza virus infections, as are studies aimed to better understand the biology and virulence factors of this significant human respiratory pathogen. Moreover, new approaches to attenuate the virus are urgently needed to overcome the limitations associated with the current LAIV.

Public health concerns associated with influenza viral infections in humans are intensified by the limited therapeutic options to combat viral infections and prevent the rapid-spreading of influenza viruses [50]. Up to now, only a few therapeutic choices to control influenza infections are licensed and they are limited to two classes of FDA-approved antivirals targeting the viral matrix 2 (M2) ion channel protein (amantadine or rimantadine) or the sialidase active site of the viral NA protein (oseltamivir, zanamivir, or peramivir) [51,52,53,54]. M2 inhibitors are only efficient against IAV, and they have shown serious side effects and low efficacy since most of the currently circulating IAV have acquired resistance to them [55]. On the other hand, NA blockers are efficient against IAV and IBV, but the emergence of drug-resistant strains is currently increasing [31,56,57].

## 2. IAV Genome Organization, Virion Structure, and Life Cycle

Although influenza viruses shar similar structural characteristics and comparable replication and transcription mechanisms, in this review we will focus on IAV and the study of its polymerase complex to develop novel prophylactic strategies against IAV infections.

IAV contains a single-stranded negative-sense viral (v)RNA genome consisting of eight segments that are encapsidated within enveloped spherical or filamentous particles (Figure 1A) [18]. The IAV genome encodes up to 16 different viral proteins using multiple strategies such as overlapping open reading frames (ORFs), alteRNAtive splicing and frameshift mechanisms [18,58,59,60]. Each of the vRNA segments are flanked at both termini by non-coding regions (NCRs) that serve as polymerase promoters for viral genome replication and gene transcription (Figure 1B). Structurally, the anti-parallel double helices of vRNAs are coated with the viral nucleoprotein (NP) (Figure 1A) to form viral ribonucleoprotein complexes (vRNPs) [61]. The vRNPs contain a polymerase complex at one end that pseudo-circularizes the vRNA [62,63,64]. Although influenza vRNAs are single-stranded, the last nucleotides at their 5′ and 3′ ends are partially complementary and are effectively circularized by being bound to the polymerase complex [65]. The IAV polymerase complex is a heterotrimeric structure of approximately 270 kilodaltons (kDa) that contains three protein subunits: the polymerase basic 2 and 1 (PB2 and PB1, respectively) and acidic (PA) proteins that, together with the viral NP, allow each vRNP to act as an independent transcription-replication element in infected cells (Figure 1C) [62,66,67].

The viral HA, NA, and M2 are structural proteins located in the virion membrane (Figure 1A). Influenza viral transcription and replication occurs in the nucleus of infected cells using a unique mechanism among negative-sense RNA viruses [68]. Upon the release of vRNPs into the cytoplasm of infected cells, vRNPs are transported into the nucleus via a process mediated by the viral NP. Once in the nucleus, the vRNA is used as a template for either transcription into capped and poly-adenylated messenger (m)RNAs [69], or for replication into complementary (c)RNA (Figure 1C). New synthetized polymerase PB2, PB1, and PA subunits, and NP are assembled to form cRNPs and the progeny vRNPs, which are produced through replication of the cRNA intermediate. While PB2, PB1, and NP contain nuclear localization signals (NLS), the PA subunit has to be associated with the viral PB1 for its importation into the nucleus of infected cells. Newly generated vRNPs are transported from the nucleus to the cytosol, a process mediated by the nuclear export protein (NEP) and the matrix 1 (M1) protein, and to the plasma membrane where vRNPs are encapsidated in a lipid envelope derived from the infected host plasma membrane. To this end, M1 interacts with the cytoplasmic tails of HA, NA, and M2 and with the membrane itself [70,71,72,73]. Release of newly synthesized virions requires removing SA from glycans on the cellular surface and the newly formed budding virions, a process mediated by the viral NA. Freely, progeny viruses are then able to start a new replication cycle by infecting new target cells [70,71,72,73].

## 3. IAV Polymerase Complex and RNA Synthesis

The analysis of viral polymerases and their crystal structures have drastically improved our knowledge of viral infection, replication, and pathogenesis. Importantly, the polymerase of IAV has been shown to also be important for virus adaptation to new host species and it is involved in the emergence of zoonotic IAV strains [10,74,75,76,77]. Moreover, since viral polymerases are important for replication and transcription of the viral genome, they represent excellent targets for the development of novel antivirals. The study of IAV polymerase structure has been challenging until recently since researchers were not able to express the intact viral heterotrimer polymerase complex (PB2, PB1, and PA) maintaining its catalytic activity. However, this drawback has been partially solved by studying the structure of the polymerase complex from bat IAV (H17N10), IBV, and ICV [13]. The bat IAV and IBV polymerases similarity to human (or avian) IAV polymerase is 70–80% and 30–40%, respectively [78]. In addition, the recently resolved crystal structure of the polymerase complex with the vRNA promoter from bat IAV [63] could increase our current understanding of the mechanism driving viral polymerase activity or the interaction between the different polymerase subunits and the vRNA and/or cellular host factors [61,79,80]. Remarkably, there are high similarity between the determined polymerases structures with bound promoters for bat IAV and IBV [63,78,81,82]. Importantly, this new knowledge will also allow researchers to rationally develop attenuated forms of the viruses that could be implemented as potential LAIV for the treatment of influenza viral infections. Likewise, information obtained from the viral polymerase complex structures would allow the rational design of novel antivirals targeting specific polymerase functions and/or components for the treatment of influenza viral infections [78,83,84].

The replicase complex core of IAV is formed by the PB1 polymerase subunit together with the C-terminal domain of PA and the N-terminal 35 residues of PB2. This complex has a polymerase core element similar to those of other viral RNA-dependent RNA polymerases (RdRp), although overall it is a much larger enzyme complex with several structured domains outside of this core [85,86]. IAV carries out the RNA synthesis in two phases (Figure 1C). In a first phase, the influenza viral polymerase complex catalyzes the transcription of vRNA into mRNA using primers generated by the PA subunit from host capped RNAs. For that, the vRNPs associate with the cellular DNA-dependent RNA polymerase II [62,67,78,83]. In a second step, cRNA are generated in a primer-independent mechanism using vRNA as template, and the synthesized viral cRNAs then serve as templates to produce new molecules of vRNA [62,81,87,88] (Figure 1C). The generated cRNA and vRNA are packaged into new cRNPs or vRNPs, respectively. It has been reported that, during the early stages of viral infection, there is a dominance of mRNAs transcription, with a minority of cRNAs, while at later phases of infection the synthesis of vRNAs increases and becomes the most abundant RNA in infected cells [62,67,78,83]. Various hypotheses have been proposed to explain the mechanism that regulates the switch from viral gene transcription to viral genome replication and the recent crystal structure of the viral polymerase will be decisive to understand the machinery of influenza RNA synthesis. The three subunits of the polymerase complex (PB2, PB1, and PA) are multifunctional proteins that carry out the viral genome replication and gene transcription in perfect coordination by regulating their cellular localization and protein–protein interactions.

### 3.1. PB1 Subunit

**Figure 2:**
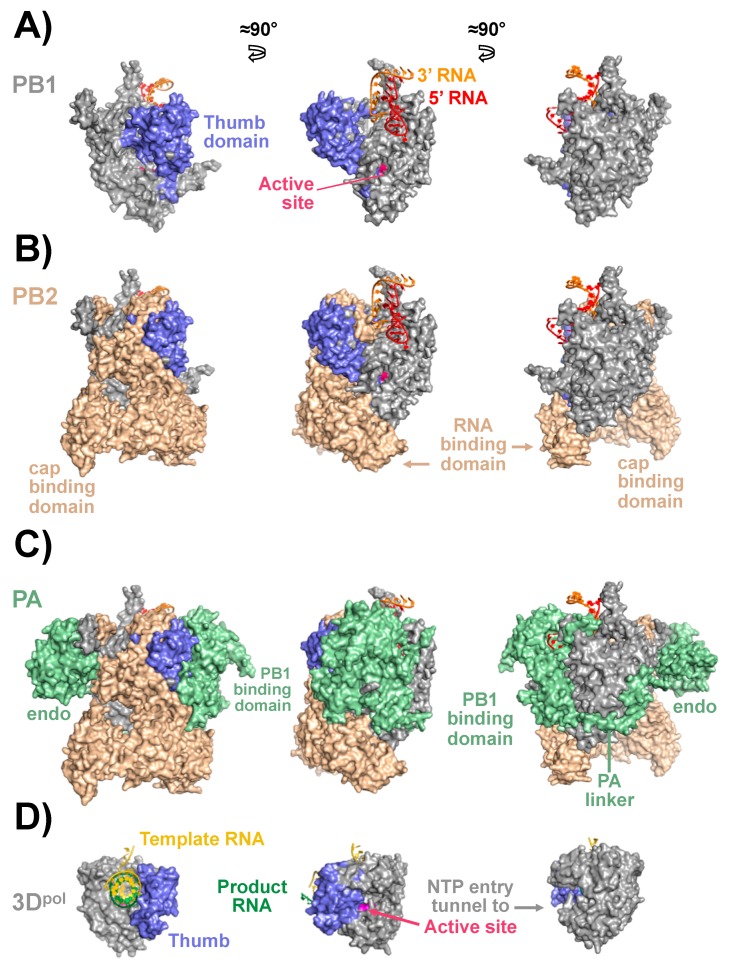
Structure of the IAV polymerase complex. The IAV polymerase complex structure is shown in three different views (left to right) rotated by ≈90° between each, and the same coloring is retained in all images (PDB code 4WSB). A dynamic version of this figure where the molecules can be rotated in real time is available online as Figure S1. Panel A shows the PB1 RdRp subunit in grey with its thumb domain colored blue, the active site within conserved motif C shown in magenta, and the 5′ and 3′ RNAs shown in red and orange, respectively. Panel B adds the PB2 subunit in wheat color with its two major domains (RNA and cap binding) indicated. PB2 associates with a large surface on PB1, effectively forming one large globular structure from which the PB2 cap binding and RNA binding domains protrude. Panel C illustrates how the RdRp PA subunit wraps around the PB2-PB1 complex, with its N-terminal endonuclease domain on one side of the complex, the PB1 binding domain forming the major exterior surface of the complex on the other side, and the linker sequence being extended across the PB1 surface. Panel D shows the to-scale structure of the poliovirus polymerase elongation complex, among the smallest single subunit viral polymerases, for size comparison and in the same orientations (PDB code 3OL6).

**Figure 3:**
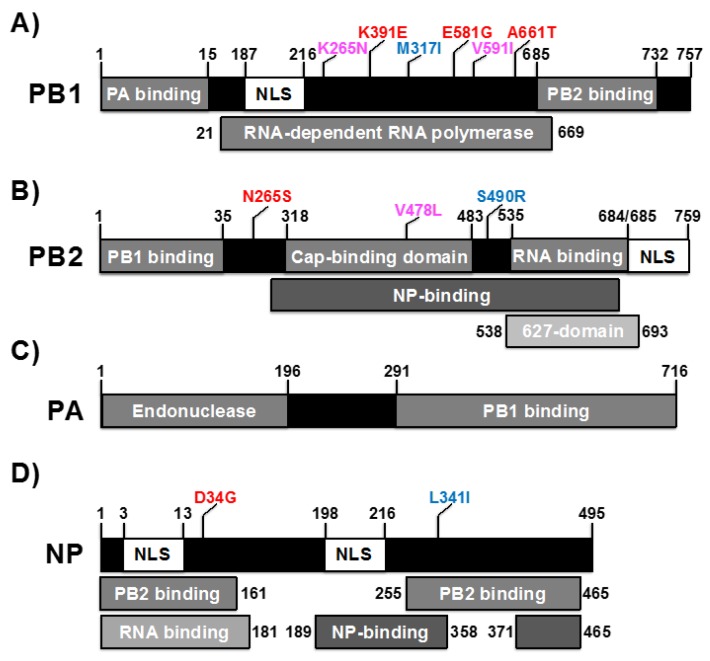
Schematic representation of the IAV virus polymerase complex protein subunits. Graphical illustration of IAV PB1 (**A**), PB2 (**B**), PA (**C**), and NP (**D**), showing the location of the conserved functional domains. Amino acid substitutions responsible for the temperature sensitive (ts), attenuated (att), and cold-adapted (ca) phenotype of the MDV LAIV A/Ann Arbor/6/60 (PB1: K391E, E581G, and A661T; PB2: N265S; NP D34G), Len/17 (PB1: K265N and V591I; PB2: V478L), and Len/47 (Len/47 substitutions and additional amino acid changes in PB1: M317I; PB2: S490R; NP: L341I) are indicated in red, magenta, or blue, respectively. NLS, nuclear localization signal. Figure not to scale.

The influenza PB1 component contains the RdRp catalytic site and is the core of the viral polymerase complex (Figure 2A). Similarly to others viral RdRp, the structure of IAV PB1 has the characteristic right hand fold, containing fingers, palm, and thumb domains that include the classic pre-A (or F) and A-E polymerase motifs (Figure 2A), although IAV RdRp is bigger than other viral polymerases (compare Figure 2A for IAV RdRp and Figure 2D for poliovirus polymerase) [13,62,67,78,83,85]. These conserved polymerase regions have been mapped in the central region of PB1 (Figure 3A). However, IAV PB1 needs the cap-binding action of PB2 and the endonuclease activity of PA to carry out viral mRNA synthesis [87,89], making the whole transcriptase complex significantly larger than the core RdRp that is often contained within a single protein in other RNA viruses. IAV PB1 possess N- and C-terminal domains that interact with PA and PB2, respectively, to stabilize this complex (Figure 3A). Moreover, PB1 contains a NLS (Figure 3A), although it has also been observed that PB1 depends on an interaction with IAV PA for its nuclear import [90]. Importantly, because the unique characteristics of the viral RdRp and the crucial role of PB1 for RNA synthesis, IAV PB1 is a promising target for the generation and identification of novel antivirals to treat influenza infections [62,91].

### 3.2. PB2 Subunit

The main role of IAV PB2 polymerase subunit is the production of 5′-capped RNA molecules from cellular pre-mRNAs, which are used as primers to initiate viral transcription [80,87]. PB2 contains multiple functional domains necessary to perform viral RNA synthesis (Figure 3B). The N-terminal domain of IAV PB2 contains the PB1 interacting region (Figure 2B and Figure 3B) while the C-terminal domain contains an RNA binding domain and a NLS for nuclear import from the cytoplasm (Figure 2B and Figure 3B) [82]. Upon infection, PB2 is located mainly in the nucleus, although PB2 has also been identified in the mitochondria [92,93]. The C-terminal two-thirds of IAV PB2 contains the cap-binding domain (CBD) and the “627-domain” [94,95] (Figure 2B and Figure 3B). Within the CBD, two aromatic residues (F404 and H357) are involved in the binding to the cap structure for the cap snatching process in which a cellular mRNA is cleaved a few nucleotides after the 5′ cap, similarly to other cellular cap-binding proteins, such as the eukaryotic initiation factor 4E (eIF4E) [87,96]. It has been reported that the PB2 subunit is an important determinant of virulence and a host range restriction of IAV and this characteristic has been mapped mainly to amino acid at position 627 and the surrounding region [97,98] (Figure 3B). Avian IAV contain a glutamic acid (E) at position 627 while human IAV have a lysine (K) [76]. Although the role of this residue for host adaptation is not totally understood, it has been described that PB2 binding to the cellular nuclear protein ANP32A could be involved in the mechanism of host adaptation [99]. Moreover, Nilsson et al. have recently showed that the 627-domain is essential for the accumulation of the cRNA replicative intermediate in infected cells and this domain is also essential for both viral replication and transcription [94]. However, other amino acid residues located in other viral genes have also been described to be important for virus host adaptation for IAV [76,94]. PB2 is also able to bind NP and the amino acids involved in this interaction are located in the middle and C-terminal region of IAV PB2 (Figure 3A) [62,67,78,83].

### 3.3. PA Subunit

To perform the transcription from vRNA to mRNA, IAV needs to generate primers. IAV polymerase does not have capping activity and it relies on cellular capped RNAs as cap-donors. This process is known as cap-snatching. First, the PB2 CBD binds the nascent host capped RNAs and then the viral PA cleaves the capped RNA using its endonuclease activity [81,87], and RNA synthesis is then completed by the RdRp activity of the IAV PB1 subunit (Figure 2C and Figure 3C) [62,78,100]. The IAV PA subunit is divided into two main domains, an N-terminal endonuclease domain [89,100] and a large C-terminal PB1-binding domain that is tightly co-folded on the PB1 subunit (Figure 2C). The endonuclease catalytic site is similar to other type II endonucleases and contains a two divalent cation dependent active-site that coordinates Mn^2+^ or Mg^2+^ using the catalytic residues H41, E80, D108, and E119 that are also conserved among IAV [101,102]. Because the distinctive characteristics of the IAV endonuclease, multiple compounds have been identified that can act as endonuclease inhibitors [101,103]. The C-terminal region of PA is responsible for the binding to the PB1 subunit (Figure 2C) and it was shown that IAV PA and PB1 form a dimer in the cytoplasm, which is then imported into the nucleus to associate with PB2 and form the heterotrimeric polymerase complex [67,83,104,105].

### 3.4. NP

IAV NP is a multifunctional basic protein essential for the replication of the viral genome and its transcription, which in crystal structures forms a trimeric complex [106]. While the IAV polymerase heterotrimer complex (PB2, PB1, and PA) is able to synthesize short RNAs, NP is required for the processivity of the polymerase on long RNA templates [107,108,109,110,111]. NP contains an RNA-binding region at its N-terminus region (Figure 3D) and two domains responsible for NP-NP self-interaction, at the center and C-terminal region (Figure 3D), although phosphorylation of NP could also play a role in its oligomerization [112]. In addition, it has been shown that IAV NP also binds through its N-terminal and C-terminal region to the viral polymerase subunit PB2 [96,113], suggesting a potential role in the regulation of the viral polymerase activity. Besides the structural functions of IAV NP as component of the viral cRNP or vRNP, NP also facilitates vRNP import into the nucleus through its two NLS located at the N-terminal and central regions (Figure 3D) [61,112,114,115,116]. Finally, IAV NP’s interaction with the viral M1/NEP complex is required for the translocation of vRNPs from the nucleus to the cytosol of infected cells at the latest steps of the replication cycle of IAV [61,112,115,116].

## 4. Temperature Sensitive (ts) Mutations in IAV Polymerase

Currently, vaccination represent the best prophylactic measure to protect against viral infections. Multiple strategies and technology approaches have been developed for the generation of vaccines with safety profiles and immunogenic and protective characteristics. Several viral LAV are associated with a ts phenotype [38,117] and they have been outstandingly successful in controlling infections by multiple viruses [118,119,120]. It is likely that ts mutations attenuate viruses by diminishing viral replication or pathogenesis at a specific body temperature, while maintaining the immunogenicity of a wild-type (WT) virus. Therefore, the use of ts attenuated viruses are considered as effective LAV and one of the best prophylactic measure to prevent disease caused by viral infections, including influenza. Moreover, in the case of influenza, LAIV administration mimics the route of a natural infection inducing mucosal immunity, which is important to inhibit viral infectivity in the host upper respiratory track at the initial steps of viral infection [121]. Consequently, the development of novel strategies that increase the immunogenicity and protection efficacy of current LAIV approaches with a similar, or higher, safety profile are extremely desired [38]. Here, we will review well-known ts mutations in the viral polymerase of IAV, and further discuss the potential use of the resolved crystal structure of the IAV polymerase complex for the rational design of novel LAIV candidates to protect against IAV infections.

## 5. Live Attenuated Influenza Vaccine (LAIV)

**Figure 4:**
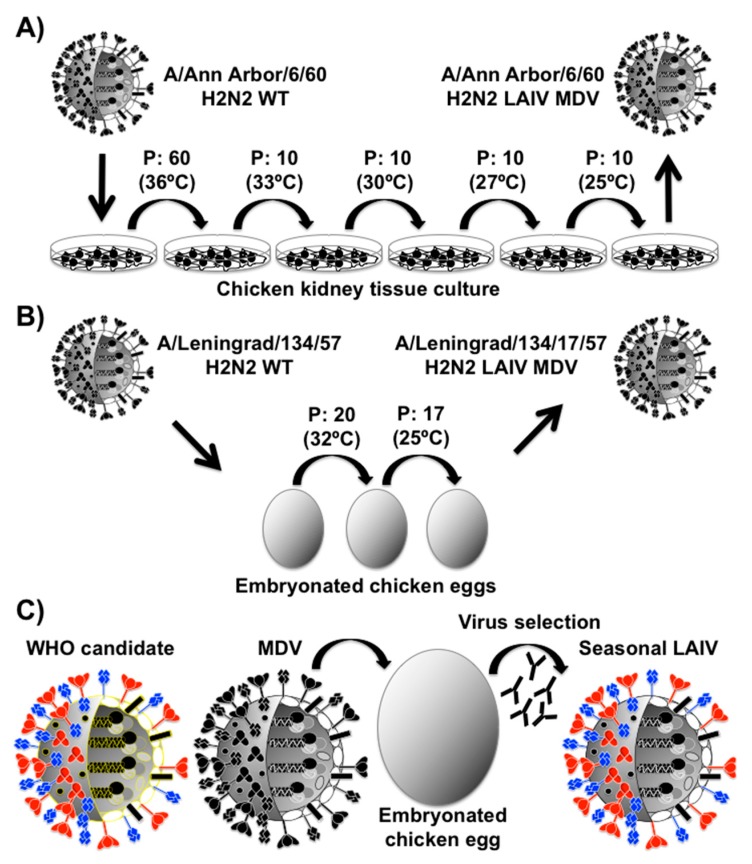
Generation of LAIV: (**A**) Isolation of the A/Ann Arbor/6/60 H2N2 LAIV MDV: A/Ann Arbor/6/60 H2N2 wild-type (WT) was serially passage 100 times (P1 to P100) in chicken kidney tissue culture (CKTC) cells under gradually reduced temperatures (36 °C to 24 °C). The obtained A/Ann Arbor/6/60 LAIV contained several mutations and the ones responsible for the att, ca, and ts has been mapped to a single residue in PB2 (N265S), three residues in PB1 (K391E, E581G, and A661T) and a single residue (D34G) in NP (Figure 2). The A/Ann Arbor/6/60 H2N2 LAIV is used as master donor virus (MDV) to produce the seasonal LAIV. (**B**) Generation of the A/Leningrad/134/17/57 H2N2 LAIV MDV: A/Leningrad/134/17/57 H2N2 WT was serially passage 20 times (P1 to P20) in embryonated chicken eggs at optimal temperature of 32 °C. Then, the A/Leningrad/134/17/57 H2N2 LAIV MDV was obtained after 17 additional passages at 25 °C. (**C**) Schematic representation to produce the seasonal LAIV: The traditional method for generating reassortant virus is based on the co-infection with two influenza viruses in eggs. Both the WHO candidate IAV (left) and the A/Ann Arbor/6/60 H2N2 LAIV MDV (right) are inoculated in eggs followed by the selection of appropriate seed viruses by amplification in the presence of antibodies against the HA and NA of A/Ann Arbor/6/60 H2N2. The resulting virus containing the HA (red) and NA (blue) segments from the WHO-recommended IAV strain and the six inteRNAl vRNAs of the MDV A/Ann Arbor/6/60 H2N2 LAIV is amplified and used for vaccine production and used as a seasonal LAIV.

The human LAIV used in the US was generated by the adaptation of an IAV (A/Ann Arbor/6/60 H2N2) to growth at suboptimal decreasing temperatures (36–25 °C) during multiple passages in primary chicken kidney tissue culture (CKTC) cells (Figure 4A) [120,122,123,124]. During this ts adaptation process, A/Ann Arbor/6/60 H2N2 gained multiple mutations in the viral genome, which are responsible of the ts and cold-adapted (ca) phenotype that allow the virus to grow efficiently at low (25 °C) temperatures. However, viral replication is restricted at higher temperatures, which are found in the human lower respiratory tract or during febrile process (>35 °C), resulting in an attenuated (att) phenotype [120,122,123,124]. This allows the host to mount an effective immune response to the infection in the upper airway and nasal cavity, without risking pathogenesis from a lower airway infection, which is the site of the most significant sequelae of influenza infection [28,36].

Current seasonal LAIV can be generated either by classical reassortment in embryonated chicken eggs or by the newly developed plasmid-based reverse genetic techniques [38,125]. These contain the six inteRNAl genes (PB2, PB1, PA, NP, M, and NS) from the original ts, ca, att master donor virus (MDV) A/Ann Arbor/6/60 H2N2 and the two viral glycoproteins (HA and NA) from predicted circulating seasonal IAV (Figure 4B).

**Figure 5:**
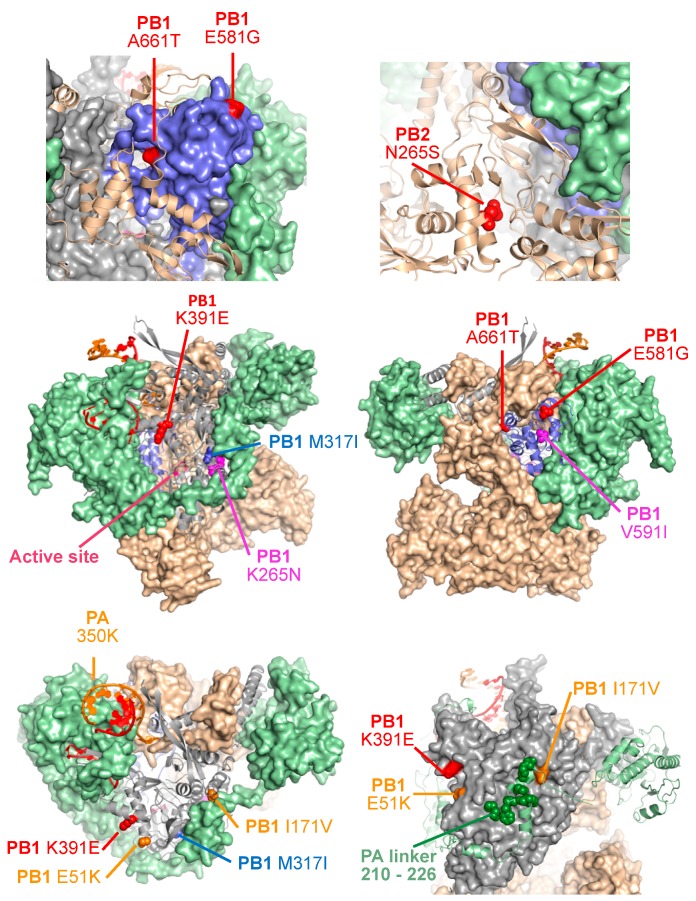
Attenuation and reversion mutations in the IAV polymerase complex. Attenuation mutations in the IAV polymerase: The locations of various IAV mutations found in the MDV LAIV and in revertant strains are shown as CPK spheres on the background of the polymerase complex colored as in Figure 2, with some subunits being shown as surfaces and others as cartoons in various orientations as needed for clarity. A dynamic version of this figure where the molecules can be rotated in real time is available on-lines as Figure S2. Mutations in the US LAIV MDV A/Ann Arbor/6/60 H2N2 are shown in red. Mutations present in the Russian LAIV MDV Len/17 or Len/47 (A/Leningrad/134/57 H2N2) strain are shown in magenta or blue, respectively. PB2 mutations V478L (Len/17) and S490R (Len/47) are not indicated in the structure. For more information see Figure 2 and Table 1. A set of engineered mutations in the PA subunit linker segment that wraps around PB1 are shown in green. Reversion mutations in PB1 (E51K, I171V) and PA (N350K) that restore virulence in the US LAIV MDV A/Ann Arbor/6/60 H2N2 (Table 2, green) are shown in orange.

The mutations responsible for the ts, ca, and att phenotype of IAV MDV A/Ann Arbor/6/60 H2N2 used in the US have been mapped in three viral segments: the viral polymerase subunits PB1 (K391E, D581G, and A661T) and PB2 (N265S) (Figure 3 and Figure 5; and Table 1, red); and the viral NP (D34G) (Table 1, red) [38,118,120,123,126,127]. Interestingly, in the Union of Soviet Socialist Republics (USSR), two different ts, ca, att IAV MDVs derived from the same virus strain (A/Leningrad/134/57 H2N2) were generated and have been used for the preparation of seasonal LAIV [128,129,130]. A/Leningrad/134/17/57 (Len/17) H2N2 MDV was obtained after 20 sequential passages at 32 °C and 17 additional passages at 25 °C of A/Leningrad/134/57 H2N2 WT in chicken embryonated eggs. A/Leningrad/134/47/57 (Len/47) H2N2 MDV was obtained similarly to Len/17 but after 47 sequential additional passages in chicken embryonated eggs at 25 °C. [129,131,132,133]. Interestingly, four different genetic changes have been involved in the ts, ca, att phenotype of the MDV Len/17 located in three viral segments: PB1 (K265N and V591I) and PB2 (V478L), NEP (M100I) (Figure 3 and Figure 5; and Table 1, magenta) [131]. Although the genetic changes responsible for the further ts, ca phenotype of Len/47 have not been well characterized, the virus contains three additional amino acid substitutions in the PB1 (M317I), PB2 (S490R), and NP (L341I), as compare with Len/17 (Figure 3 and Figure 5; Table 1, blue) [134,135]. The MDV Len/47 is more attenuated than Len/17 and has been used for vaccinating children less than 16 years of age in Russia [135,136]. However, since Len/47 MDV showed reduced immunogenicity compared to Len/17, the use of Len/47 has been discontinued [132].

The contributions of the different amino acid changes in the polymerase components of the current IAV MDV strains for the ca, ts, and att phenotype have only been partially characterized and their mechanism of attenuation is not entirely understood, but most likely involve multiple steps in the replication cycle of the virus [137]. However, it has been shown that the amino acid substitutions K391E and E581G of the MDV A/Ann Arbor/6/60 H2N2 used in the US are the major contributors for the ts and att phenotype. In the case of Len/47, the main mutations responsible for that phenotype are amino acid changes in the PB1 (K265N and V591I) and PB2 (V478L) [138]. Importantly, when the genetic signature of the US MDV A/Ann Arbor/6/60 H2N2 was introduced into of the backbone of other human IAV such as A/Puerto Rico/8/34 H1N1 (PR8) [126], A/California/04/09 H1N1 (pH1N1) [103,139] or canine (A/canine/NY/dog23/09 H3N8) [118,127] and equine (A/equine/Ohio/03 H3N8) [140] influenza viruses, a similar ts and att phenotype was observed in tissue culture cells as well as in an animal models of IAV infection (Table 1) [103,118,126,127,139,140,141]. Significantly, the current commercially available ts, ca H3N8 EIV LAIV, which was developed by passaging the A/equine/Kentucky/1/91 H3N8 in embryonated chicken eggs at gradually reduced temperatures [142,143] have not been updated to match the currently circulating EIV strain. Furthermore, it has been observed that there is not an optimal match of surface antigens between circulating EIV strains and the virus present in the vaccine, leading to a suboptimal protection [144,145,146]. Moreover, the mutations responsible for the ts, ca, and att phenotype of EIV LAIV have not been yet characterized. However, when we introduced, using reverse genetics approaches, the genetic signature of the US MDV A/Ann Arbor/6/60 H2N2 in a more recently circulating EIV strain (A/equine/Ohio/1/03 H3N8) we were able to generate a new and updated EIV LAIV for the prevention and control of currently circulating H3N8 EIV in horse populations (Table 1) [140]. These data further demonstrate the contribution of the identified amino acid residues in the human US MDV A/Ann Arbor/6/60 H2N2 in the ts, ca, and att phenotype but also suggest a common approach to generate new and more efficient LAIV to protect not only humans but also other animals from the devastating effects of IAV infections.

## 6. Safety Profile of LAIV

There are multiple scientific and clinical evidences demonstrating the safety profile of the licensed seasonal human US LAIV (FluMist). However, recently Zhou et al. carried out an interesting study to evaluate the ability of the attenuated US LAIV to revert the ts and att phenotype in vitro [156]. Intriguingly, authors have reported that the original attenuated MDV might revert to a virulent virus after 20 passages (P1-P20) in vitro at gradually elevated temperatures from 30 °C to 39 °C. Moreover, they were able to identify seven amino acid substitutions responsible for the reverting phenotype (Table 2, green; Figure 5) [156]. The role of these individual mutations was analyzed at various temperatures by evaluating their effects on RNA polymerase activity and virus replication [156]. Authors found that only five amino acid changes: PB1 (E51K and I171V; found in P10 and P15, respectively), PA (N350K; found in P5), and NP (N125Y and/or V186I; found at P5 and P20, respectively) were enough to revert the ts phenotype of the US MDV A/Ann Arbor/6/60 H2N2. Importantly, in vivo, the revertant virus was pathogenic in mice as compared to the MDV A/Ann Arbor/6/60 H2N2. Interestingly, the amino acid changes responsible for the reversion phenotype were not mapped in positions previously defined to be responsible of the ts, ca, and att phenotype of the MDV A/Ann Arbor/6/60 H2N2 (Table 1 and Figure 5, red).

Tsfasman et al. studied a revertant virus for the MDV Len/47, which was generated after 18 passages at elevated temperatures (up to 40 °C) in embryonated chicken eggs [133]. During this adaptation, the virus lost the ts and ca phenotype, since the virus was able to grow with high titers at 40 °C, and also the ability to replicate efficiently at lower temperatures (26 °C). Most importantly, the revertant virus replicated at similar levels than the WT A/Leningrad/134/57 H2N2 virus in mouse lungs. Sequencing of the revertant virus discovered 29 nucleotide changes, 15 of them resulting in amino acid substitutions in PB2 (L478V), PB1 (T156I, N265K, K358E, D521A, and Q686E), PA (D327E, Q452H, and V463A), NP (D101N and A180G), M1 (F144L and S231D), and NS1 (P23A and P164L) (Table 2, magenta). Sequencing confirmed the reversion of two ts mutations in the PB2 (L478V) and PB1 (N265K) genes. However, the effect for the rest of amino acid substitutions in other viral genes was not evaluated [133].

It is important to highlight that all the described revertant mutants have been generated under laboratory conditions. However, after vaccination, pathogenic revertants from the current MDVs used in the human LAIVs has been not observed, demonstrating that they have a very tested safety profile. Interestingly, the majority of amino acid substitutions identified in both revertant mutants are located in the polymerase complex, indicating that polymerase genes are most crucial in the ts phenotype maintenance of the MDV LAIVs.

## 7. Location of Revertant and ts Mutations in the Polymerase Structure

By using the recent resolved crystal structure of the bat IAV polymerase (PDB ID 4WSB) or A/WSN/33 H1N1 (WSN) NP (PDB ID 3RO5), the location of the identified amino acid variations in the revertant or original ts viruses can be analyzed (Figure 5). In PB1, the original ts, ca, and att amino acid change A661T localize in a region where PB1 and PB2 interact and in the PB1:RNA interface (Figure 5). In addition, the amino acid substitutions K391E and E51K in the PB1 from the original MDV A/Ann Arbor/6/60 H2N2 or the revertant viruses, respectively, are far apart in the linear sequence, although they appear to be proximal in the protein structure (Figure 5) [156]. Moreover, there is a basic (K) to acidic (E) change in the original MDV A/Ann Arbor/6/60 H2N2 PB1 at position 391 (Figure 5), while the described revertant has an acidic (E) to basic (K) change in the same structural region at position 51, suggesting that this region may be involved in interactions with other viral and/or host cellular proteins (Figure 5) [156]. Interestingly, with the exception of NP I186, the amino acid changes in the MDV A/Ann Arbor/6/60 H2N2 revertants were mapped on the surfaces of the PB1 (K51 and V171), PA subunits (K350), and NP (Y125) (Figure 5). PB1 V171 is located in the interface between PB1 and PA (Figure 5), and this position could be involved in intermolecular PB1-PA interactions. In fact, the amino acid substitution N350K identified in the PA polymerase subunit of the revertant MDV A/Ann Arbor/6/60 H2N2 virus could also be involved in this interaction with the PB1 subunit (Figure 5) [156].

The PB2 N265S amino acid substitution in the MDV A/Ann Arbor/6/60 H2N2 appears to be part of electrostatic interactions between two PB2 domains, which might explain the ts phenotype associate to this change (Figure 5). However, many details regarding the location and potential functions of amino acids involved in ts, ca, and att phenotypes remain elusive because the lack of high-resolution structures depicting the interactions between the polymerase subunits or their interaction with the viral NP. Therefore, it is difficult to conclude the role of these amino acid substitutions. Moreover, it is possible that the mutations in the viral polymerase complex or NP responsible for the ts, ca, and att phenotype of the MDV A/Ann Arbor/6/60 H2N2 affect known or unknown interactions with other viral and/or host cellular proteins.

## 8. Other Amino Acid Mutations in the IAV Polymerase Associated with a ts Phenotype

In addition to the mutations located in the viral segments of the current licensed human LAIV, genetic studies from multiple researchers using different IAV strains have identified other amino acid changes in the viral polymerase complex (PB2, PB1, and PA) and NP that are related with a ts and att phenotype [120,123,131,157,158,159,160,161]. The analysis of all these amino acid substitutions is out of the scope of this review, and this section is limited to provide some examples with relevance for vaccine design and/or development. However, it is important emphasize that high resolution influenza polymerase structure will have significant implications to understand the mechanisms involved in ts and att phenotypes.

Efficient nuclear import and assembly of the polymerase subunits (PB2, PB1, and PA) are necessaries steps during viral replication. Da Costa et al. used circular dichroism analysis to study the PA linker structure of WSN, showing that it is a structurally disordered domain [150]. Moreover, they reported that some of the PA linker mutants (T210P, K213P, D216P, L219P, L219A, P221A, N222P, F223P, S225P, and L226P) exhibited a ts phenotype (reduced viral growth at 39.5 °C versus 37 °C or 33 °C) (Table 1), which was suggested to be a consequence of altered folding kinetics [150]. Moreover, using a minigenome system, the ts phenotype of the PA mutants T210P, K213P, D216P, F223P, and L226P was associated with a reduced efficiency in viral replication/transcription [150]. Immunofluorescence assays also suggest an inefficient recruit of PB1 to the nucleus at restrictive temperatures (39.5 °C) when co-expressed with PA mutants L214P or D216P, L219P, or F223P. Importantly, some of the ts viruses (D216P and L219P) were attenuated in a mouse model of infection and D216P was still able to induce strong humoral response, suggesting that this strategy could be used to develop novel IAV LAIV candidates. Interestingly, revertants for the ts phenotype were selected by serial passages of the ts mutants at 39.5 °C. Authors identified three groups of reversions (Table 2, black): changes in other amino acids of the PA protein (E377K), reversions at the residue that was initially mutated, or amino acid substitutions in other components of the viral polymerase complex such as PB2 (R136G) or PB1 (R287M). Therefore, the isolation of revertant mutants in ts viruses allowed the identification of compensatory changes located in one of the three-polymerase subunits, which could be important to understand the interactions between the components of the viral polymerase complex [150]. These data demonstrate the importance of studying the IAV polymerase complex to understand the mechanism behind the ts and ca phenotypes in order to design improved LAIV approaches. Furthermore, the study of potential revertant viruses could assist researchers in elucidating the mechanism of viral replication and transcription as well as the mechanism(s) of attenuation. Most importantly, the genetic stability of ts LAIV should be closely evaluated as part of the att safety profile of new potential IAV LAIV.

Avian IAV are an emerging epidemiological concern. For instance, highly pathogenic avian H5N1 IAV (HPAIV) are continuously circulating in many Asian countries and threatening poultry industry [162]. Although HPAIV do not normally infect humans, some H5N1 strains can cause severe infections in humans with lethality rates of up to 60% [162]. Hence, prophylactic vaccination approaches are the best option to control H5N1 HPAIV infections in poultry and to prevent their transmission to the human population. Zhang et al. generated a recombinant reassortant virus (C4/W1) that contained the NA gene and a modified HA gene from A/chicken/Hubei/327/04 H5N1 (C4), and the six inteRNAl genes from the MDV A/duck/Hubei/W1/04 H9N2 (W1) [149]. The recombinant virus was subsequently passaged in chicken embryonated eggs at progressively lower temperatures (32, 28, and 25 °C). Authors isolated a ts, att C4/W1 virus that provided efficient protection in chickens against H5N1 HPAIV challenge [149]. Importantly, they identified a mutation in the PA polymerase subunit (F35S) (Table 1) that was associated with the ts phenotype. These findings demonstrate the potential use of new identified ts and att viruses as well as their respective mutations in the development and implementation of new LAIV to protect against IAV infections, as well as to prevent important economy loss or potential pandemic risks.

IAV NP is a multifunctional protein that is involved in vRNA nuclear import, viral replication and transcription, and particle encapsidation [61,112]. Moreover, the role of NP in the adaptation of IAV to a new host has been reported [163]. NP amino acid changes have also been involved in ca and/or ts phenotypes [120,123,137,164]. Pulkina et al. studied an IAV MDV A/Hong Kong/1/68/162/35 H3N2, which was adapted to growth at low temperature [151]. The new virus contains 14 amino acid changes in the inteRNAl proteins. Two of those mutations were mapped in the NP (G102R and E292G) and using reverse genetics technologies, the authors generated recombinant viruses containing these amino acid substitutions [151]. Then, the viral replication of those viruses was evaluated at different temperatures in embryonated eggs. Reassortants with the reverse mutation G292E in the viral NP were characterized by non-ca and ts phenotype. The introduction of the second reverse mutation R102G, in the context of G292E, completely abolished the virus ca phenotype. Intriguing, viruses with single reverse mutation at amino acid position 102 in NP failed to be rescued [151].

Similarly, Noton et al. described the characterization of an avian influenza virus (A/chicken/Rostock/34 H7N1) ts NP mutant (M239L) (Table 1) [152]. This mutant virus was generated by chemical mutagenesis in the 1970s. Importantly, introduction of this ts NP M239L amino acid change in the backbone of PR8 reproduced the ts phenotype [152]. Despite a 100-fold drop in viral titers at the restrictive temperature (39 °C), the PR8 NP M239L ts mutant virus did not show differences in gene transcription, genome replication and/or viral protein synthesis as compared with PR8 WT virus [152]. However, the ts mutant PR8 NP M239L virus released about six-fold more virus particles than the WT PR8. Interestingly, the viral particle/plaque forming unit (PFU) ratio of the ts mutant PR8 NP M239L was 50-fold higher than that of PR8 WT, with numerous virions exhibiting a nonstandard morphology [152]. Authors revealed that the NP ts mutation M239L affects the NP-M1 interaction and most probably the assembly of viral particles [152]. Importantly, this study demonstrates that ts IAV can be affected in one or more viral life cycle steps, from viral RNA synthesis to virus morphogenesis and release. Moreover, this study further confirms that ts amino acid substitutions in a specific IAV strain could display a similar ts phenotype when introduced in a different IAV backbone [38,118,126,127,140].

Recently, Jang et al., using a previously developed ts and ca X-31 H3N2 IAV [147], analyzed the genetic signature of the virus and characterized the contribution of the identified amino acid changes in the ts, ca phenotype of the IAV X-31 [148]. This mutant virus was selected during multiple serial passages in embryonated eggs at progressively lower sub-optimal temperatures (30, 27, and 24 °C). The cold-passaged mutant exhibited both ts and ca phenotypes. Moreover, mice did not show clinical signs even at high titer infection with the generated ts and ca X-31 mutant virus [147]. X-31 is a reassortant virus carrying the HA and NA segments of A/Hong Kong/1/68 H3N2 in the genetic background of PR8 [126,147]. During the ca process, 32 nucleotide changes were introduced in all six inteRNAl viral genes. Among them, 17 nucleotide substitutions resulted in amino acid substitutions in seven viral proteins: four in PB2 (F34Y, M105I, H110Q, and I588S), three in PB1 (V91A, A240T, and G684E), two in PA (S69N and I668V), two in NP (E18G and T130M), three in M1 (F3L, A137T, and I217R), two in M2 (M42I and A86T), and one in non-structural protein 1 (NS1; M98L) (Table 1) [147,148]. Phenotypic analysis with single- and multiple-gene reassortant viruses suggests that the viral NP was the major contributor to the ts and ca phenotype of the ts and ca X-31 H3N2 IAV [147]. However, other viral genes also included mutations responsible for viral attenuation in mice [147]. The contribution of the six inteRNAl genes from most to least to the ca phenotype were NP > PB1 = M > PA > PB2 = NS and that to the ts phenotype were NP > PB2 = PA > M > NS > PB1 [147]. In addition, multiple gene reassortant viruses containing combinations of the polymerase components (PB2, PB1, and PA) provided marginal effect to the ca or ts phenotype as compared to the ts and ca phenotype of the single NP gene [147]. Moreover, by combining the three polymerase subunits (PB2, PB1, and PA) with the NP gene, the authors observed an increase in both phenotypes (ca and ts) [148], suggesting an additive effect of several amino acid residues in the polymerase complex. However, in this study, authors did not identify the specific amino acid residues in each of the viral proteins involved in the ca, ts, and att phenotype.

## 9. Temperature Sensitive Mutations Associated to Other Viral Segments

Although the IAV viral machinery for RNA synthesis represents an important target to introduce mutations to generate LAIV with a ts, ca, and att phenotype, it is feasible that amino acid changes in other viral proteins could also be similarly considered and explored for the development of new ts, ca, and att LAIV [131,155,160].

M1 is a structural protein of the IAV virion that has multiple functions during viral infection. The dissociation of M1 from vRNP is required for the entry of vRNPs into the cytoplasm of the host cell during initial infection. Moreover, the association of IAV M1 with vRNA and NP is a step indispensable for the formation of vRNP and their nuclear export [165,166,167,168]. Liu et al. introduced several amino acid substitutions in the backbone of A/WSN/33 to understand the role of the zinc finger motif and the RKLKR domain of M1, both of them involved in RNA binding, during viral assembly and replication [153]. They observed that modifying the zinc finger motif of IAV M1 reduced viral growth slightly [153]. On the other hand, by introducing the amino acid substitutions R101S or R105S in the RKLKR domain of IAV M1, a reduction for both the nuclear export of vRNP and viral replication was observed [153]. Interestingly, the double IAV M1 mutant R101S-R105S, displayed a ts phenotype (39.5 °C) (Table 1) [153]. Moreover, the virus containing both M1 substitutions (R101S-R105S) had a reduced ratio of M1 to NP in viral particles and a weaker binding of M1 to RNPs [153]. Importantly, both substitutions were required to prevent the nuclear localization of M1 at the restricted temperature, while single amino acid substitutions at either position had a minimal effect on the nuclear localization of the viral M1. Likewise, Nogales et al., have shown how the split of the M segment (Ms) of PR8 to produce M1 and M2 from a single transcript rather than alteRNAtive splicing, results in a virus with a ts and att phenotype able to protect against lethal challenge with WT PR8 [126,169]. Notably, protection efficacy of the modified Ms PR8 virus was better than that obtained with a ts, ca PR8 [169], demonstrating the feasibility of using this split approach of the M segment, alone or in combination with the viral NS, for the development of safe, immunogenic, and protective LAIV [169].

The defense mechanisms provided by the host innate immune system restrict IAV infection [170]. Therefore, to efficiently replicate in interferon (IFN)-competent systems, IAV has to regulate the production of IFNs and the activities of IFN-induced proteins that inhibit virus replication [171]. IAV NS genome segment encodes NS1, which is a multifunctional protein mainly involved in counteract host innate immune responses, modulating virus pathogenesis. Interestingly, it has been described that some mutations in the NS1 protein can confer a ts phenotype to IAV by different mechanisms. In fact, IAV mutants expressing deletions and/or truncations of NS1 show deficiencies in replication at non-permissive temperature (39 °C) [38,155,172,173,174].

Garaigorta et al. identified a ts mutant (named 11C) of A/Victoria/3/75 H3N2 that contained three amino acid changes in the NS1 protein: V18A, R44K, and S195P, which were responsible for the ts phenotype [154]. The replication and gene expression of the 11C virus was only slightly altered [154]. However, the mutations had a high impact in virus particle formation, suggesting that NS1 could be involved in the morphogenesis of IAV [154]. Interestingly, when these mutations were introduced in the backbone of PR8 [126], the PR8-11C virus was most attenuated at 39 °C compared to WT PR8, consistent with previous results [154]. Moreover, a recombinant PR8 virus containing the mutations responsible for the ts, ca, and att phenotype of the MDV A/Ann Arbor/6/60 H2N2 (PB2 N265S and PB1 K391E, D581G and A661T) alone (PR8 LAIV) or in combination with the 11C mutations in NS1 (PR8 LAIV-11C) [126] was severely impaired to replicate or make plaques at restrictive temperatures (39 °C) as compared to PR8 LAIV, which could likely be attributed to the NS1 11C mutations affecting virus egress/budding [126]. In addition, PR8 LAIV-11C showed higher attenuation in vivo than PR8 LAIV, but was able to stimulate similar humoral responses and able to protect against a lethal challenge with the WT PR8 virus than PR8 LAIV [126]. These results highlight the possibility to increasing the safety profile of the current MDV A/Ann Arbor/6/60 H2N2 LAIV without affecting its immunogenicity and protection efficacy by introducing the NS1 11C mutations [126].

Recently, the variability of the NS1 protein of circulating IAV H3N2 isolated from infected subjects was analyzed, and multiple amino acid changes were identified [155,175]. The consequences of these mutations on the NS1-mediated inhibition of IFN responses and the pathogenesis of the virus were evaluated, showing that some NS1 mutations impaired the ability of the NS1 protein to inhibit host gene expression [155,175]. Interestingly, one of the identified NS1 mutations (V194I) was associated with a ts phenotype [155,175]. Moreover, we have showed that the virus encoding the NS1 V194I mutation was attenuated in vivo as compared to a virus containing the WT NS1 protein [155,175]. These studies are relevant, not only to generate novel LAIV, but also in order to identify new residues essential for NS1 functions, including the identification of mutations that can be used as risk assessment of circulating pathogenic viruses in the human population [155,175].

## 10. Conclusions

During the last decade, seasonal IAV vaccines have shown a low effectiveness in protecting humans against annual viral infections [34,38,45]. There are multiple reasons than can affect the efficiencies of LAIV or IIV. (i) The route of administration. LAIV are administered by the intranasal route, mimicking a natural infection, and can establish mucosal immunity in the respiratory tract. On the other hand, IIV are administered intramuscularly, leading to suboptimal induction of immune responses in the mucosa [45,176]. (ii) The intrinsic immunogenicity of the vaccine components, mainly the HA protein, can be responsible for the differences observed in the efficacy of seasonal IIV and/or LAIV [177]. (iii) The emergence of viral strain variants, distinct from the selected vaccine strain, during a given influenza season can result in significant loss of vaccine effectiveness. (iv) The establishment of influenza-specific B- and T-cell memory [178]. Since the MDV of the IAV LAIV remains constant between seasons, and only the viral HA and NA genes are updated, it has been suggested that preexisting immunity to the inteRNAl proteins of the MDV could limit the response to the LAIV, which must replicate in order to be immunogenic and provide protection against subsequent viral infections. In fact, this could be one of the reasons for the low efficacy of LAIV. Understanding how seasonal IAV vaccines are influenced by preexisting immunity will be important for developing the next-generation of IAV vaccines. Moreover, futures strategies to develop new vaccines against IAV, which are highly desired, might require modifying the MDV A/Ann Arbor/6/60 H2N2 currently used for the preparation of seasonal and pandemic LAIV.

The recently published high-resolution structures of the IAV polymerase complex and the new advances in the study of the mechanism of vRNA synthesis have expanded our current knowledge of this threatening human respiratory pathogen, providing researchers the opportunity to analyze the mechanism of virulence, transmission, and/or host adaptation. In addition, understanding the mechanism(s) of regulation of the viral polymerase complex could assist in the rational design of safer and more immunogenic and protective LAIV. In this review, we have discussed the use of ts, ca, and att mutations in IAV for the generation of LAIV. This approach could provide a deliberate method of viral attenuation, which could be applicable for the treatment of either seasonal and/or pandemic IAV infections, or viral strains infecting other host species. Furthermore, combination of polymerase thermosensitivity with other approaches can assure a higher attenuated profile for the development of safer LAIV as well as advance in the generation of universal IAV vaccines [35,38,58,103,126,179,180]. Universal vaccines candidates are required to induce cross-protective broadly neutralizing immunity, stimulating both humoral and cellular adaptive immune responses. Moreover, universal vaccines should avoid the necessity of annual re-vaccination [37,41]. Given that LAIV typically induce a stronger antibody and T-cell mediated responses and mucosa protection, they represent an excellent option as a universal vaccine platform [37,41]. Finally, insights into the molecular mechanisms of viral replication and transcription could provide important information for the rational structure-based design of ts, ca, and att LAIV as well as the identification of novel antivirals for the prevention and treatment, respectively, of IAV infections.

## Figures and Tables

**Table 1:** Amino acid mutations in IAV polymerase complex associated with a temperature sensitive phenotype. Amino acid substitutions of the US MDV A/Ann Arbor/6/60 H2N2 (red), the Russian MDV A/Leningrad/134/17/57, Len/17 (magenta) and the Russian MDV A/Leningrad/134/47/57, Len/47 (blue) H2N2 LAIVs are indicated (for more information see Figure 3).

GeTne	Amino Acid Mutation	Backbone	Reference
PB2	N265S	A/Ann Arbor/6/60 H2N2 A/Puerto Rico/8/34 H1N1 A/California/04/09 H1N1 A/canine/NY/dog23/09 H3N8 A/equine/Ohio/03 H3N8	[103,118,120,123,126,127,131,139,140,141]
PB2	V478L	A/Leningrad/134/17/57 H2N2; Len/17	[131,134,135]
PB2	S490R	A/Leningrad/134/47/57 H2N2; Len/47	[131,134,135]
PB2	F34Y, M105I, H11, I588S	X31	[147,148]
PB1	K391E, E581G, A661T	A/Ann Arbor/6/60 H2N2 A/Puerto Rico/8/34 H1N1 A/California/04/09 H1N1 A/canine/NY/dog23/09 H3N8 A/equine/Ohio/03 H3N8	[103,118,120,123,126,127,131,139,140,141]
PB1	K265N, V591I	A/Leningrad/134/17/57 H2N2; Len/17	[131,134,135]
PB1	M317I	A/Leningrad/134/47/57 H2N2; Len/47	[131,134,135]
PB1	V91A, A240T, G684E	X31	[147,148]
PA	T210P, K213P, D216P, L219P, L219A, P221A, N222P, F223P, S225P, L226P	A/WSN/33 H1N1	[149,150]
PA	F35S	A/duck/Hubei/W1/04 H9N2	[149,150]
PA	S69N, I668V	X31	[147,148]
NP	D34G	A/Ann Arbor/6/60 H2N2 A/Puerto Rico/8/34 H1N1 A/California/04/09 H1N1 A/canine/NY/dog23/09 H3N8 A/equine/Ohio/03 H3N8	[103,118,120,123,126,127,131,139,140,141]
NP	L341I	A/Leningrad/134/47/57 H2N2; Len/47	[131,134,135]
NP	G102R, E292G	A/Hong Kong/1/68/162/35 H3N2	[151]
NP	M239L	A/chicken/Rostock/34 H7N1 A/Puerto Rico/8/34 H1N1	[103,118,120,123,126,127,131,139,140,141,152]
NP	E18G, T130M	X31	[147,148]
M1	R101S, R105S	A/WSN/33 H1N1	[153]
M1	F3L, A137T, I217R	X31	[147,148]
M2	M42I, A86T	X31	[147,148]
NS1	V18A, R44K, S195P	A/Victoria/3/75 H3N2 A/Puerto Rico/8/34 H1N1	[126,154]
NS1	V194I	A/Puerto Rico/8/34 H1N1	[155]
NS1	M98L	X31	[147,148]
NEP	M100I	A/Leningrad/134/17/57 H2N2; Len/17	[131,134,135]

**Table 2:** Amino acid mutations responsible for the reversion of US MDV A/Ann Arbor/6/60 H2N2 LAIV (red), the Russian MDV A/Leningrad/134/47/57, Len/47 (blue) and WSN (black) to a pathogenic phenotype.

Gene	Amino Acid Mutation	Reference
PB1	E51K, I171V	[156]
PA	N350K	[156]
NP	N125Y, V186I	[156]
PB2	L478V	[133]
PB1	T156I, N265K, K358E, D521A, Q686E	[133]
PA	D327E, Q452H, V463A	[133]
NP	D101N, A180G	[133]
M1	F144L, S231D	[133]
NS1	P23A, P164L	[133]
PB2	R136G	[150]
PB1	R287M	[150]
PA	E377K	[150]

## Supplementary Materials

The following are available online at http://www.mdpi.com/1999-4915/10/10/560/s1, Figure S1: Dynamic version of Figure 2. Figure S2: Dynamic version of Figure 5. The dynamic versions of these figures allow the reader to rotate and zoom the polymerase structures in real time in a normal web browser window. The supplemental figures have multiple ‘scenes’ that correspond to the panels shown within each printed figure, and the same figure legends apply.

## Abbreviations

ACIP	Advisory Committee on Immunization Practices
Att	Attenuated
Ca	Cold-adapted
CBD	Cap-binding domain
CEIRS	Centers of Excellence for Influenza Research and Surveillance
CIV	Canine influenza virus(es)
CKTC	Chicken kidney tissue culture
cRNA	Complementary RNA
eIF4E	Eukaryotic initiation factor 4E
EIV	Equine influenza virus(es)
FDA	Food and Drug Administration
HA	Hemagglutinin
HPAIV	Highly pathogenic avian influenza virus(es)
IAV	Influenza A virus(es)
IBV	Influenza B virus(es)
ICV	Influenza C virus(es)
IDV	Influenza D virus(es)
IFN	Interferon
IIV	Inactivated influenza vaccine(s)
kDa	Kilodalton
LAIV	Live attenuated influenza vaccine(s)
LAV	Live attenuated vaccine(s)
Len/17	A/Leningrad/134/17/57 H2N2 LAIV
Len/47	A/Leningrad/134/47/57 H2N2 LAIV
M1	Matrix protein 1
M2	Matrix protein 2
MDV	Master donor virus
NA	Neuraminidase
NAbs	Neutralizing antibodies
NCR	Non-coding region
NEP	Nuclear export protein
NIAID	National Institute of Allergy and Infectious Diseases
NIH	National Institutes of Health
NLS	Nuclear localization signal
NP	Nucleoprotein
NS1	Non-structural protein 1
NYICE	New York Influenza Center of Excellence
ORF	Open reading frame
PA	Polymerase acid protein
PB1	Polymerase basic protein 1
PB2	Polymerase basic protein 2
PFU	Plaque forming units
pH1N1	Pandemic A/California/04/09 H1N1 influenza virus
PR8	A/Puerto Rico/8/34 H1N1 influenza virus
RBD	Receptor binding domain
RdRp	Viral RNA-dependent RNA polymerase
SA	Sialic acid
Ts	Temperature-sensitive
vRNA	Viral RNA
vRNP	Viral ribonucleoprotein
US	United States
USSR	Union of Soviet Socialist Republics
WHO	World Health Organization
WSN	A/WSN/33 H1N1 influenza virus
WT	Wild-type
X31	Reassortant influenza virus containing the HA and NA genes of A/Hong Kong/1/68 H3N2 in the background of influenza PR8 virus
